# Isolation Huts

**Published:** 1886-12-25

**Authors:** 


### ISOLATION HUTS: AN INQUIRY FROM AMERICA.

One of the most active workers in the Hospital field in America writes to us from New York as follows:—

"Can The Hospital advise an American correspondent on the subject of isolating huts in connection with a small suburban general hospital, where ground space is ample, but where the winter climate is severe? The best plan known to the writer is that of a gable-roofed wooden cottage of one room, twenty feet square, giving space for two patients, with a third bed for a nurse; the latter screened by a slight partition. This hut is raised on wooden or brick posts above an asphalt pavement which extends under and round the building. The hut has double walls and floor; the interspace between walls being ventilated by swing-boards on the outside, while between the flooring large tin pipes are laid, with registers in the floor near the beds. These floor pipes run into a drum or outer casing of a warm smoke-pipe behind a stove, and carry off foul air from the level of the floor. The stove, having an open hearth, promotes ventilation directly as well as indirectly. Fresh cold air is admitted by swing-sashes over the door and windows, and by other openings, with scatter-plates at the level of the eaves. Such a cottage has been in successful use for ten years on the grounds of the Presbyterian Hospital, New York city, a somewhat sheltered spot. But your correspondent desires to know whether there is anything in England of more recent construction and better plan."

[We have received the following interesting particulars from Mr. D. Keith Young, 17, Southampton Street, W.C., the eminent hospital architect, who has devoted so much time and attention to these matters:—"Wooden huts have been in use at some of the Metropolitan Fever Hospitals as extra wards for epidemic periods. In such cases they are usually constructed in the manner described by 'an American correspondent,' but the warming is effected by hot-water pipes running round the wards at the floor level; fresh air is admitted at the level of the pipes by openings in the enclosing walls; the foul air is extracted at the ridge, either by cowls or by lowered openings, and fresh air is admitted also by the windows on opposite sides of the wards. These huts, however, not having been erected as permanent buildings, are not constructed in as careful a way as they should be if intended to last for any lengthened period. In a very cold or exposed situation, the enclosing walls should be formed in much the same way as 'half-timber work,' that is, there should be three layers either of wood or plaster, and a space of at least $1\frac{3}{4}$ inches between the layers, and the space between the inner lining and the centre layer should be filled in with asbestos, or some other equally non-conducting substance. The roof should be treated in a similar fashion, so that the ward would be completely surrounded by an enclosed cushion of air, with an inner layer of non-conducting substance."]

